# Design of Sports Rehabilitation Training System Based on EEMD Algorithm

**DOI:** 10.1155/2022/9987313

**Published:** 2022-06-06

**Authors:** Kaiwei Wang, Zhenghui Wang, Wu Ren, Chunsheng Yang

**Affiliations:** ^1^School of Physical Education, Xinxiang Medical University, Xinxiang, Henan 453003, China; ^2^Pukyong National University, Busan 608-737, Republic of Korea; ^3^The First Affiliated Hospital of Xinxiang Medical University, Xinxiang, Henan 453003, China; ^4^College of Medical Engineering, Xinxiang Medical University, Xinxiang, Henan 453003, China; ^5^Rehabilitation Department, The Third Affilitated Hospital of Xinxiang Medical University, Xinxiang, Henan 453003, China

## Abstract

Motor function rehabilitation training is to restore the motor function of hand injury to the maximum extent and meet the needs of patients' daily behavior. At present, motor function evaluation and rehabilitation training work have disadvantages such as relying on the subjective experience of physicians, unable to quantitatively assess the loss of motor function, and single rehabilitation training method. Most of these methods only focus on the independent motion range of a single organ, lack of consideration of the constraint relationship between adjacent fingers, and do not build a visual model for it. To end this issue, for the purpose of sports rehabilitation, combined with the status and application of rehabilitation machines, this paper proposed a cycling rehabilitation training system based on physiological signal extraction of ensemble empirical mode decomposition (EEMD) algorithm. Results compared with the previous rehabilitation training, the muscle tension level of patients' upper limbs decreased, and the strength of some muscles also increased. With the progress of rehabilitation training, the contralateral dominance coefficient showed an upward trend, which further confirmed the role of the proposed method in sports rehabilitation, and also provided a new idea for the evaluation of rehabilitation training effect of patients in the future.

## 1. Introduction

According to the data of the sixth national census in 2010, the population aged 60 years and over accounted for 13.26% of the total population in China, among which the population aged 65 years and over accounted for 8.87%, up 2.93 percentage points and 1.91 percentage points, respectively, compared with 2000. With the rapid development of population aging, many countries in the world have also entered the aging society. In modern society, with the increase of the number of elderly people, the health status and disease care of the elderly are more and more concerned by the society [1]. On one hand, it is necessary to grasp the physical state of the elderly in real time, so as to make timely prevention and treatment measures according to the changes of their physical signs [2, 3]. On the other hand, there is bound to be a large number of patients with diseases in the elderly, especially those with severe and chronic diseases, which requires real-time monitoring of the health status and rehabilitation of the elderly [4].

Long ago in our country, many institutions have been carried out on lower limb rehabilitation robot research, the purpose is to gradually replace the traditional medical division as the core of rehabilitation. According to the different degree of rehabilitation of lower limb movement disorders, lower limb rehabilitation robot can provide incremental training mode, from auxiliary training to active rehabilitation training [5]. The lower limb rehabilitation robot system can use the acquisition – perception system to record the law of human movement. Extraction movement characteristics of lower limb rehabilitation training in order to provide effective data to support the intelligent control system design of multisensor information fusion technology not only provides lower limb movement disorder patients in rehabilitation training monitoring and protection but can also identify for lower limb movement disorder gait movement patterns, thus better supporting leg movement disorders increase rehabilitation exercise capacity [6, 7]. At present, people's evaluation of finger rehabilitation degree is mostly achieved by hand function rehabilitation assessment scale and finger joint range of motion to achieve hand function rehabilitation assessment scale mostly through moving objects, such as pinch and stretch hand function rehabilitation assessment. The range of motion of finger is evaluated by measuring the range of motion of finger joint. The traditional measurement method of finger range of motion mostly uses angle meter to measure the range of motion of finger joint. With the development of science and technology, many researchers began to use mobile phones to measure the range of movement of fingers. However, the measuring method is paid more attention to the hand refers to the movement range of flex movement, overlook the fingers stretch and abducent movement within range. According to the study found that the evaluation method is more focused on the hand movement function recovery, rehabilitation evaluation lack of quantitative indicators for a single finger. In addition, most of the traditional measurement data read directly by the people, prone to error, and the computer can accurately obtain the finger motion range according to the positioning and tracking, and reduce the error.

Many clinical patients have motor dysfunction to a certain extent. How to restore their motor skills has always been a difficult problem in the field of rehabilitation training. However, traditional rehabilitation training methods are often unable to fully mobilize the enthusiasm of patients, and there are safety risks, such as hand arm rehabilitation training is easy to cause new injuries due to collisions, and patients cannot complete complex movements through the mechanical arm to adapt to daily life. The results show that a large number of repetitive and high-density rehabilitation training can maximize the recovery of patients' physical functions, improve their quality of life, and improve their ability to live, work, and study. The traditional rehabilitation training method is mainly carried out by auxiliary massage, which is required to be carried out in professional locations [8, 9]. The training and treatment process are monotonous, and the training effect and training intensity cannot be accurately evaluated. In order to overcome the shortcomings of the traditional rehabilitation training method, a large number of researchers have found through studies that active lower limb rehabilitation exercise can improve the impaired motor function of patients. Rehabilitation training to improve the ability of independent living refers to the purpose of improving the patients' lower limb motor ability, according to different stages of rehabilitation to choose the appropriate exercise mode, the corresponding rehabilitation exercise lower limb rehabilitation training machine is one of the main means of patients to restore motor function, is widely used in rehabilitation training. Lower limb rehabilitation training system by simulation of lower limb movement situation, improve the state of lower limb muscles, promote the recovery of lower limb movement function, design and development is more suitable for patients with lower limb rehabilitation training system can greatly enhance the recovery rate of cerebral apoplexy patients, easing the burden on the family and society, to improve the level of social medical treatment and improve the people's health level is of great significance.

At present, hand robots, data gloves, and virtual reality technology are mostly used for hand rehabilitation [10–14]. Hand robot is mainly through the robot to drive the hand to grasp and flex the movement of hand rehabilitation training. Data gloves and virtual reality technology mainly model the hand first and then realize the rehabilitation training of the hand through hand movement, such as virtual hand grasping, holding, moving objects, and throwing objects. These methods have achieved remarkable results in hand function rehabilitation training, but pay more attention to the whole hand motor function rehabilitation training, lack of single organ motor function rehabilitation training methods. In addition, these methods cannot fully reflect the finger movement state and rehabilitation degree. Therefore, the research topic of individual organ rehabilitation training is more necessary and has more practical significance. The evaluation of rehabilitation degree of motor function is of great significance to finger rehabilitation training. Therefore, researchers attach great importance to the evaluation of rehabilitation degree of motor function. At present, the degree of rehabilitation is evaluated by functional rehabilitation scale and range of motion. However, these measurement methods pay more attention to the range of motion of flexion and extension, but ignore the range of motion of extension and abduction. According to the research findings, these assessment methods pay more attention to the rehabilitation of the whole motor function and lack of quantitative indicators for individual rehabilitation assessment. In addition, most of the traditional measurement data are read directly by people, easy to produce errors, but the computer can accurately obtain the motion range according to positioning and tracking, and reduce the error.

Based on the above discussion, the main contributions of this paper are given as below:

1. We designed a cycling rehabilitation training system based on physiological signal extraction of ensemble empirical mode decomposition (EEMD) algorithm.
2. The muscle tension level of patients' upper limbs decreased and the strength of some muscles also increased.
3. We provided a new idea for the evaluation of rehabilitation training effect of patients in the future.

## 2. Related Work

The traditional rehabilitation monitoring is in rehabilitation doctor handy or with the help of a simple instrument to assist patients complete the process of rehabilitation training, in the process of rehabilitation training rehabilitation doctor based on their experience of the patient's physical condition after training the stand or fall of the degree and the rehabilitation effect of evaluation, the monitoring and evaluation method is largely dependent on been physician experience, level and state, low efficiency, have greater limitations. Some teams have carried out researches on home nursing and remote health monitoring and developed the home intimate little nurse system. The system is composed of a home monitor and a hospital console. The home monitor can record the ECG, EEG, and dynamic blood pressure of patients in the process of rehabilitation in real time [15]. The hospital monitoring console can receive and display the ECG, EEG, and blood pressure data of multiple home users at the same time and can return the diagnosis opinions to home users. The development of wireless communication, especially short-range wireless communication technology, makes wireless network become a new method to design and implement rehabilitation monitoring system [16–20].

The nodes developed in the wireless monitoring system were significantly improved in mobility compared with wired physiological monitoring. However, there are still many deficiencies in comfort and convenience. In order to solve the above problems, people begin to combine the emerging wearable technology and wireless sensor network technology and gradually apply it to the design of physiological parameter monitoring system. Wireless body area network is a new wireless communication technology developed in recent years, which combines the advantages of wearable and wireless sensor network, and has become one of the frontiers and hotspots in the field of information research. Limb rehabilitation robots are military exoskeleton research and application based on the exoskeleton of features should be used on the rehabilitation medicine helps lower limbs dyskinesia patients with rehabilitation training, lower limbs dyskinesia patients go CCB ability. In recent years, some research institutions launched a lower limb rehabilitation robot product, but because of the lack of research on sensor technology information fusion technology and machine learning, the functions of rehabilitation robot products are still not perfect. The rehabilitation training system can adjust the pull value of the rope and change the size of the patient's lower limb support force according to the force of patients with different lower limb movement disorders. The system by stimulating the quadriceps femoris two receptors and adjust the patient's gait and posture, so that the patients completely simulate the normal gait trajectory, so as to help patients in the cerebral cortex to rebuild or rebuild the walking posture functional area with the help of the system, according to the rehabilitation degree of patients with lower limb movement disorders to choose the corresponding recovery pattern, make exercise treadmill lead the limb implement step the passive training and training, and other rehabilitation mode, provide more suitable for patients with lower limb dyskinesia rehabilitation plan, can also be suitable for more of the lower limbs dyskinesia patients rehabilitation training, at the same time, the system can timely feedback to the effect of rehabilitation training rehabilitation evaluation system, to the patients. The effect of complex training for health evaluation and identification of abnormalities and other functions.

Sensor signals in recent years have been widely applied in the extraction and state recognition of human movement information, through the installation of different functional rehabilitation robot motion sensor's patients data, to identify different human body movement patterns to achieve higher recognition rate and more motion pattern recognition, many researchers around the feature extraction method research. However, from a single common in sensor signal can only identify a limited number of sports mode, switch signal recognition results only as a robot, so multiple sensor information fusion become another hot point in the robot technology, it can be partial data provided by the comprehensive analysis of multiple sensor information, keep the cross department between sensors. Divide and remove the conflicting information between them, based on sensor data between complement each other, reduce the uncertainty information systems, human-computer complete description of the consistent state can be formed, thus improve the robot intelligent decision-making and rapid response performance of industrial robot information fusion method including estimate class methods, such as mobile robot information theory method and statistical method artificial intelligence methods. In addition, machine learning has been widely used in various fields [21–25], by giving a series of selected features, using machine learning models, such as linear discriminant analysis (LDA), support vector machine (SVM), and K Nearest Neighbor (KNN).

However, there are few or even no rehabilitation monitoring systems for rehabilitation training occasions of stroke patients. In addition, the most physiological parameters monitoring system still stays on the physiological parameter data collection, and for data processing and analysis is not in real time, in the process of rehabilitation training, to achieve the best effect of rehabilitation, real-time control of the patient's body recovery, rehabilitation doctor must accordingly adjust training mode and training intensity. Therefore, it is very practical and necessary to research and develop a monitoring system for rehabilitation training.

## 3. Design of Sports Rehabilitation Training System by EEMD Algorithm

### 3.1. The Structure of Sports Rehabilitation Training System

**Figure 1:**
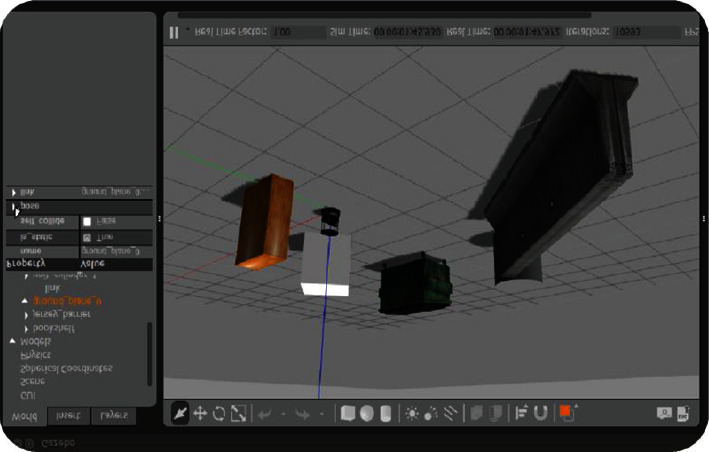
The interface of the system designed in this paper for sports rehabilitation.

The system designed in this paper is a platform capable of 3D robot kinematics and dynamics simulation and the interface of the system designed in this paper for sports rehabilitation, which is shown in Figure 1, and this platform can simulate various expected characteristics of articulated robots in complex realistic environments. The system has the following characteristics: (1) based on the powerful ODE (open dynamic engine) physics engine, the system has high-quality image rendering effect. (2) Open source and free, with convenient program and graphic design interface. (3) Achieve the test of robot control algorithm with the minimum cost. Quickly find and solve the possible problems in the real operating environment and carry out rehabilitation training. Based on the interface of the system designed in Figure 1, the subsequent research of this paper is the system framework designed based on the diagram.

Compared with traditional hand function evaluation and rehabilitation training, the functional evaluation and rehabilitation training system based on EEMD has prominent advantages, which are embodied in the objectivity of evaluation process and results, the interest of rehabilitation training, the improvement of the efficiency of medical resource allocation, and cost saving, etc. Specific analysis is as follows: all evaluation indexes are expressed in the form of joint angle values, and the quantified evaluation results can more objectively reflect the patient's hand motor function, and also help to compare the evaluation results with each standard index to find out the specific problems of motor dysfunction. On one hand, the physical movement skills learned by users in the virtual environment can be better transferred to real life; On the other hand, virtual games can greatly stimulate the initiative and enthusiasm of patients to participate in rehabilitation training. On the one hand, this system is based on the ROS distributed architecture and publishers—subscriber information communication mechanism, for evaluation and rehabilitation training data exchange and storage convenience, rehabilitation trainer can prompt understanding through the network to the patient's recovery and give guidance, shortens the recovery period, improve the efficiency of rehabilitation treatment. On the other hand, patients can conduct evaluation and rehabilitation training in the community street or at home, which not only solves the problem of lack of medical resources at the present stage but also greatly saves human and material resources and financial resources, avoiding patients to run repeatedly between family and hospital.

### 3.2. EEMD Algorithm

In the common empirical mode decomposition (EMD) method, obtaining a reasonable intrinsic mode function (IMF) depends on the distribution of signal extreme points. If the signal extreme points are not evenly distributed, mode aliasing will occur. For low signal-to-noise ratio and the diesel engine vibration signal are nonstationary characteristics, etc., adopt EEMD, adding white noise to decompose signals, using the uniform distribution of white noise spectrum, when the signal on consistent throughout the time-frequency spatial distribution of white noise background. After multiple averaging, the noise will cancel each other, and the result of the integrated mean can be used as the final result. In addition, EEMD decomposition method is also adaptive, which is superior to wavelet analysis:

(1) $$\begin{array}{c} n_{j}\left(t\right)\left(j=1,\ldots ,m\right),\\ h_{1}\left(t\right)=X\left(t\right)-m_{1}. \end{array}$$

If the *h*_1_(*t*) two conditions of IMF component are not met, the above steps should be repeated as the original signal to obtain the data sequence as *h*_11_(*t*), i.e.,

(2) $$\begin{array}{c} h_{11}\left(t\right)=h_{1}\left(t\right)-m_{11}\left(t\right),\\ S_{w}=\frac{1}{2}\sum_{i=1}^{n}\sum_{j=1}^{n}W_{i,j}^{w}\left(x_{i}-x_{j}\right){\left(x_{i}-x_{j}\right)}^{\text{T}}. \end{array}$$

It is going to be separated *c*_1_ from the signal *X*(*t*)

(3) $$\begin{array}{c} r_{1}\left(t\right)=X\left(t\right)-c_{1}\left(t\right),\\ r_{j-1}\left(t\right)-c_{j}\left(t\right)=r_{j}\left(t\right),\;j=2,3\ldots n,\\ c_{2}\left(i=1,\ldots ,N\right). \end{array}$$

In order to ensure that each IMF has the physical significance of amplitude and frequency modulation, the conditions are transformed into the following formula:

(4) $$\begin{array}{c} SD=\sum_{k=0}^{T}\frac{{\left[m_{1k}\left(t\right)-m_{1\left(k-1\right)}\left(t\right)\right]}^{2}}{m_{1\left(k-1\right)}^{2}\left(t\right)}. \end{array}$$

Then the classification decision values of a user's offline training model are calculated by (5) as below:

(5) $$\begin{array}{c} g_{k}\left(x\right)=-\frac{1}{2}{\left(x-{\bar{x}}_{k}\right)}^{\text{T}}{\hat{T}}_{r}\Gamma^{-1}{\hat{T}}_{r}^{\text{T}}\left(x-{\bar{x}}_{k}\right)+\mathrm{In}K-\frac{1}{2}\mathrm{In}\left[\text{det}\left(\Gamma \right)\right], \end{array}$$

(6) $$\begin{array}{c} \Gamma =\frac{\left({\hat{T}}_{r}^{\text{T}}\left[\sum_{x\in C_{k}}\left(x-{\bar{x}}_{k}\right){\left(x-{\bar{x}}_{k}\right)}^{\text{T}}\right]{\hat{T}}_{r}\right)}{n_{k}-1}. \end{array}$$

The average envelope of the last cycle is as follows:

(7) $$\begin{array}{c} m_{1\left(k-1\right)}\left(t\right) \end{array}$$

The ideal SD value should be between 0.2 and 0.3 so that the original data can be represented by the sum of the IMF component and the residual term:

(8) $$\begin{array}{c} X\left(t\right)=\sum_{j=1}^{n}c_{j}\left(t\right)+r_{n}\left(t\right). \end{array}$$

**Figure 2:**
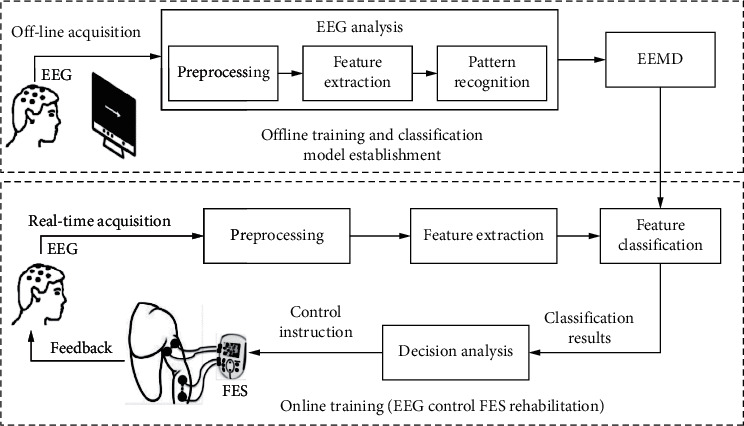
EEMD-based sports rehabilitation system structure diagram.

The designed system can be divided into four main modules (as shown in Figure 2): signal acquisition module, signal processing module, command control module, and information feedback module. The main function of the signal acquisition module is to collect the user's EEG data in real time and send it to the signal processing module block after proper processing [26]. The signal processing module sets relevant parameters, processes, and analyzes the received EEG signals, interprets the user's subjective action intention, and sends the results to the command control module. The command control module achieves the ideal rehabilitation training effect after receiving the result of subjective action intention interpretation. The information feedback module will feedback the user's limb movement state and movement intention identification results in real time to the user, activate the sensorimotor cortex, and play a positive role in promoting the patient's brain nerve function and limb movement synergistic rehabilitation and plasticity reconstruction.

## 4. Experimental Results and Analysis

### 4.1. Introduction to Experimental Environment

This line of business on Windows 11 OS runs a HMP RTX 2070S 16 GB video memory, AMD Ryzen 2400G CPU, 8 GB DDR4 software environment: deep learning framework Pytorch 1.8. Python 3.5 CUDA 10.0.

Since the system is still in the scientific research stage and has not been applied in clinical rehabilitation, samples are selected in a small range for testing and experiment of the system. In this paper, 20 subjects of different ages and genders are selected as effective samples, including 12 young people aged 15–35 years, 6 middle-aged people aged 36–59 years, and older people aged over 60 2 years, ensuring the comprehensiveness and representativeness of sample selection. Then the signals are obtained by a set of wearable devices to get the physiological signals from subjects. And we used Internet of Things to remove the movement artefact for mobile EEG and the wavelet denoising algorithm removes noise in signal, thus pure signals can be obtained for use in subsequent research.

### 4.2. Experimental Results' Analysis

**Figure 3:**
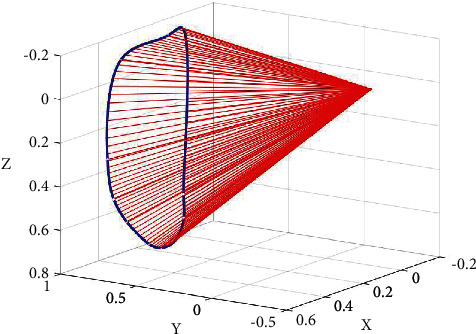
Indicates the range of motion of the ring.

First, when modeling the movement range of the ring rotation with MATLAB, the point of origin is the metacarpal joint of the finger, the direction of the parallel finger is *X*-axis, and the direction of the vertical back of the hand crossing the origin is *Z*-axis. The direction of vertical XOZ plane through the origin is *Y*-axis. Since the movement of the ring is restricted by the middle finger, the circumferential motion of the distal phalanx is not circular in specification, and the metacarpal joint of the finger moves in situ, so the circumferential motion range of the finger is similar to that of the cone using MATLAB. The range of ring turning motion is modeled, as shown in Figure 3. The graph surrounded by the blue closed curve and the red straight line is the range of ring turning motion with MCP (metacarpal joint) as the origin, which is known as the standard range of normal ring turning motion, and is used for the follow-up study of motor function rehabilitation training of injured finger.

Simple empirical mode decomposition of EEG signals will cause the problem of wide frequency band coverage, and at the same time, unsatisfactory IMFs will be produced in the low-frequency region. Signals containing low-energy components cannot be separated, which will lead to errors in the results. In order to make up for these deficiencies, EEMD will be used in EEMD. Before, the signal was divided into a group of narrowband signals by wavelet transform, and the appropriate sub-band signals were selected to decompose into the natural mode function with more concentrated frequency. The correlation coefficient of the first two orders of IMF is much higher than that of the rest of IMF. It can be seen that the first two orders of IMF contain the most important information of EEG signal, including the most active frequency band, while the rest of IMF contain noise and useless signals, so they are abandoned Therefore, in this paper, only the first two orders of IMF are selected to be reconstructed. The purpose of EMD here can be regarded as the extraction of *μ* rhythm and *β* rhythm. Meanwhile, this method can obtain more effective signals and improve the signal-to-noise ratio of the whole EEG signal.

**Figure 4:**
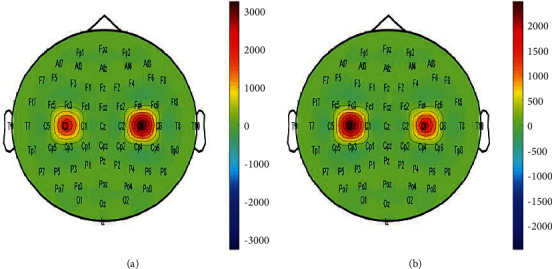
Brain topography of the first two orders of IMF.

Before using EMD, this paper extracts relevant EEG signals, selects the data of C3 and C4 channels, obtains a series of narrowband signals through WPT, and then performs EMD decomposition of corresponding sub-band signals to obtain multiple IMFs. The first two orders are selected as new inputs, forming 8 (channel) 2000 The matrix *XI*(*I* = *L* left hand, *I* = *R* right hand) selects two sub-band signals (4, 1), (4, 3), decomposes by EMD and selects the first two order IMFs. The data obtained from each test were decomposed by WPT, sub-band signals were selected, EMD decomposition was performed, IMF was selected, and the first two IMF components were combined and reconstructed. Then, CSP was used for feature extraction.  Figure 4 shows the filtered brain topography of the first two IMF components in a single experiment.

Finger horizontal rehabilitation assessment is to evaluate the rehabilitation of finger extension and abduction motor function assessment of finger extension and abduction motor function assessment is divided into static motor function assessment and dynamic motor function assessment static motor function assessment is independent motor function assessment of finger extension and abduction. Dynamic motor function assessment was used to assess the combined adduction and abduction of the index finger and middle finger.

**Figure 5:**
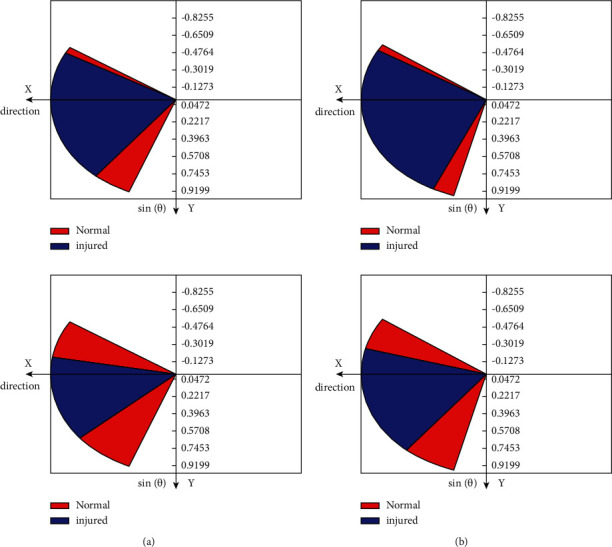
Contrast the range of motion between extension and abduction. (a) Extension, (b) abduction.

The percentage of the independent range of motion in the horizontal direction of the injured finger is the comparison of the standard range of motion in the extension and abduction of the injured finger. The comparison of the range of motion in the extension and abduction of the three patients is shown in Figure 5(a) in the extension, which, respectively, represents patient 1, patient 2, and patient 3 from top to bottom The percentage of the coordinated range of motion in the horizontal direction of the injured finger was compared with the standard coordinated range of motion in the extension and abduction of the injured finger.  Figure 5(b) shows the comparison of the coordinated range of motion in abduction of the three patients. The motion range of patient 1, patient 2, and patient 3 was compared from top to bottom, respectively.

**Figure 6:**
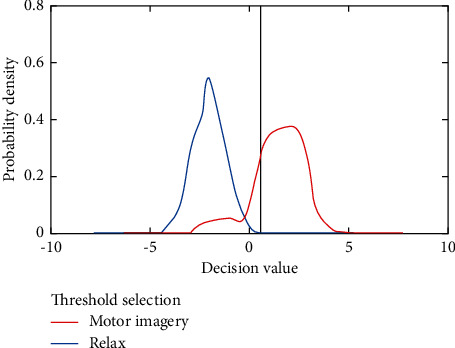
Probability density distribution of classification decision values of patient.

Considering the efficiency and accuracy of real-time operation of online system, the separability of data classification results of offline training is discussed below.  Figure 6 presents the probability density distribution curve of classification decision values which are calculated by Equation (5) of a user's offline training model, where the blue line represents the probability density distribution of classification decision values in the relaxed state mode, and the red line represents the probability density distribution of classification decision values in the visualized action mode. Effective training of patients in the correct mode of rehabilitation (simultaneous coupling of imaginary movement and electrical stimulation). The smaller the overlap area of the two curves is, the easier it is to distinguish the features of users under the two imagination task modes (relaxation and imagination action) in the actual design. Considering the rehabilitation effect of the system, this paper focuses on reducing the first type of errors of the system, namely the false positive rate. The offline training model is used to select the threshold value of decision value (to ensure that the false-positive rate is not more than 10%, that is, the area of decision value probability density curve in relaxed state is not more than 10% under the threshold value) for the threshold judgment of real-time classification decision value, so as to ensure the efficiency of real-time operation of the system, which has proved the effectiveness of the proposed method.

To test the electrical control clinical training operation performance, functional electrical stimulation recovery system to recruit two stroke patients (both for the left basal ganglia damaged cause right limbs hemiplegia) participated in the neurological rehabilitation clinical rehabilitation training test machine. As a reference, 2 healthy volunteers may also participate in the clinical test, training in the process of synchronization acquisition EEG data. In addition, in order to verify the rehabilitation effect of the system, a long-term rehabilitation training tracking was conducted for one of the patients, and the data collected during the process were analyzed for individual cases. Rehabilitation training was conducted 2–3 times a week, with each training lasting about 40 min. EEG data were collected synchronously during the experiment and the tracking process lasted for 3 months, among which the first months and 3 months for upper limb rehabilitation training (wrist and training), the second month for lower limb rehabilitation training, and record related clinical common rehabilitation rating scale. Functional electrical stimulation of rehabilitation in addition to the electrical control system, the whole process of rehabilitation training in patients with only accepted according to the traditional treatment, and no drug rehabilitation training in rehabilitation process. All 4 volunteers performed the right wrist extension exercise task.

**Figure 7:**
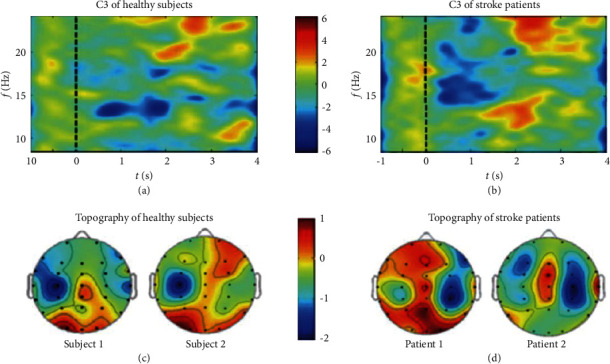
Brain topography of time-frequency characteristics in healthy volunteers and stroke patients. (a) c3 of healthy subject, (b) c3 of stoke patients, (c) topograpgy of healthy subjects, (d) topography of stoke patients.

As can be seen from Figure 7, ERD phenomenon existed in the sensorimotor cortex in both healthy volunteers and stroke patients under the image-like action mode, and some brain regions were activated. However, according to the results of brain topographic map, there was no significant difference between healthy volunteers and stroke patients was observed in healthy volunteers, while the opposite was observed in stroke patients.

**Figure 8:**
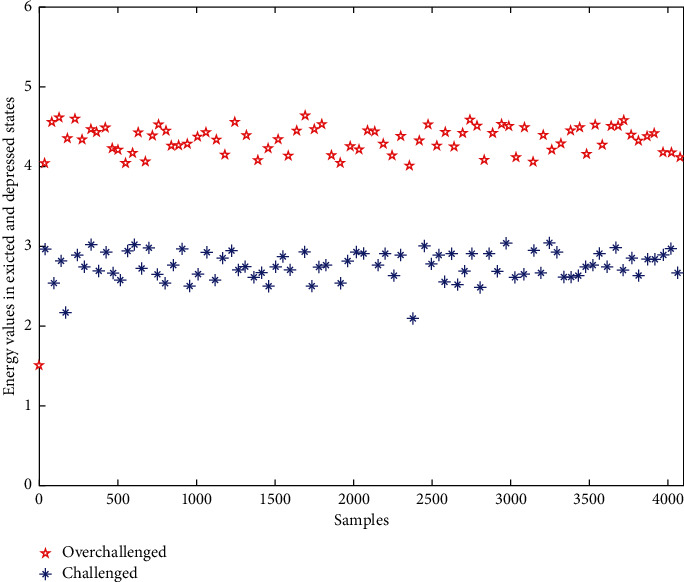
Energy profile of ECG under excited and depressed states.

In order to prove the effectiveness of the proposed method in different states of exercise rehabilitation. As shown in Figure 8, the energy profile of the electrocardiogram samples in the excited and depressed states is given to show the difference between them. As you can see from the graph, the energy displayed by the subjects undergoing either challenge training (excited state) was greater than that of the subjects undergoing overchallenge training (depressed state), with a distinct division, which shown the effectiveness of training system based on EEMD algorithm in this paper.

## 5. Conclusions

With the continuous improvement of living standards, the poor diet and the aging of the population have led to an increase in the number of patients with impaired motor function year by year, and the social burden has become increasingly heavy in order to help these patients recover or recover the motor ability of limbs to a certain extent.

In this paper, EEG signal processing methods are studied, including pretreatment, EEMD combined with WPD and CSP for feature extraction of EEG signals, and SVM method are used for pattern classification of extracted feature EEG signals, and the progress of this method is verified by experimental data. An online brain-computer interface (BCI) upper limb rehabilitation system based on motor imagination was designed, and several experiments were carried out. The system can control the robot arm in real time based on EEG signals of the experimenter and can realize the synchronization of EEG signals with the robot arm Experimental results show that the brain-controlled online arm rehabilitation system designed in this paper can fully improve the rehabilitation effect of patients with impaired motor ability.

In this paper, an online MI-BCI upper limb rehabilitation system is designed based on the research of EEMD processing method. Based on my limited knowledge and ability level, and the rapid development of EEG signal processing technology, in view of these situations, the directions to be improved in the research of this paper are envisaged: The improvement of EEG collection method and equipment can improve the accuracy of EEG collection without noise interference. However, the research in this paper is only limited to the exercise rehabilitation training system of EEG signal, and the amount of data and data types involved are small. Big data and multiple kinds of data will be the focus of future research.

## Data Availability

The data used to support the findings of this study are available from the corresponding author upon request.
